# Effectiveness of a multicenter training programme to teach point-of-care vascular ultrasound for the detection of peripheral arterial disease in people with diabetes

**DOI:** 10.1186/s13047-018-0283-0

**Published:** 2018-07-16

**Authors:** Pasha Normahani, Rishi Agrawal, Vasilliki Bravis, Agnieszka Falinska, Linda Bloomfield, Zaheer Mehar, Dawn Gaulton, Alex Sangster, Tracey Arkle, Corinna Gomm, Mohamed Aslam, Nigel J. Standfield, Usman Jaffer

**Affiliations:** 1 Imperial Vascular Unit, Imperial College NHS Healthcare Trust, St Mary’s Hospital, QEQM building, Praed Street, London, W21NY UK; 2 Department of Diabetes and Endocrinology, St Mary’s Hospital, Imperial College NHS Healthcare Trust, London, UK; 3 West Middlesex University Hospital Diabetic foot service, Chelsea and Westminster Hospital NHS Foundation Trust, Isleworth, UK; 4 Mindray Medical Ltd, Huntingdon, UK

**Keywords:** Peripheral arterial disease, Diagnosis, Diabetes, Diabetic foot, Ulcer, Duplex ultrasound, Point-of-care, PAD-scan

## Abstract

**Background:**

The primary aim of this study was to evaluate the effectiveness of a training programme to teach a focused bedside ultrasound scan (PAD-scan; Podiatry Ankle Duplex Scan) for the detection of arterial disease in people with diabetes.

**Methods:**

Five podiatrists and one diabetologist across two hospitals were enrolled in a structured training programme consisting of a training course (1-day), supervised scanning (5-weeks), independent scanning (3-weeks) and a final evaluation of performance (1-day).

Time, technical skills *(Duplex Ultrasound Objective Structured Assessment of Technical Skills tool (DUOSATS); minimum score = 6, maximum score = 26)* and accuracy (level of agreement with vascular scientist PAD-scan assessment) were assessed for every supervised scan and again for the final evaluation of performance.

**Results:**

A total of 90 PAD-scans in 65 patients were performed during the supervised phase. Participants demonstrated significant improvements in median time *(19 min(IQR 13.9–25.5)* vs *9.3 min (IQR 7.3–10.5)****;*** *p = 0.028)* and DUOSATS scores *(17.5 (IQR 16.8–21)* vs *25 (IQR 24–25.3); p = 0.027)*. At the final evaluation, participants completed scans in 5.4 min (IQR 5.3–5.9), achieved full DUOSAT scores and perfect agreement with the vascular scientist.

**Conclusion:**

A structured training programme, integrated into diabetic foot clinics, was effective in teaching the PAD-scan

**Electronic supplementary material:**

The online version of this article (10.1186/s13047-018-0283-0) contains supplementary material, which is available to authorized users.

## Background

The timely detection of arterial insufficiency in the presence of active diabetic foot disease is paramount in preventing limb loss [1, 2]. However, even in the most experienced of hands, the diagnosis of arterial disease is challenging based on clinical examination alone [3, 4]. The use of a non-invasive bedside test, such as ankle brachial pressure indices (ABPI), audible hand held Doppler waveform assessment, toe brachial pressure indices (TBPI) and transcutaneous tissue oxygen (TCO_2_), in addition to clinical history and examination is encouraged [5, 6]. However, in the presence of diabetes, no diagnostic tool alone has demonstrated sensitivity, appropriate for high stakes clinical assessment [7].

Given the severe consequences of delayed arterial disease detection, there is a need to investigate other potential bedside tests. Point-of-care vascular ultrasound may provide a feasible solution by applying readily available technology in a novel manner.

Currently, a full lower limb vascular ultrasound scan, also known as a Duplex ultrasound scan (DUS), is commonly the initial imaging modality of choice when arterial disease is suspected and has been demonstrated to have good agreement with angiography [8]. However, a full lower limb arterial scan is time consuming, challenging to learn and may not be required as a ‘rule out’ test for arterial disease. However, focused scanning of the distal anterior and posterior tibial arteries at the ankle may be able to provide information regarding the upstream state of the vasculature. We have termed this the ‘podiatry ankle Duplex scan’ (PAD-scan). The PAD-scan allows for qualitative graphical assessment of the arterial waveform to detect arterial disease.

But, in order for the scan to be feasible and immediately useful in clinical practice, it must be rapidly learned, fast and accurately performed by front line healthcare workers, such as podiatrists. Our group has previously demonstrated that podiatrists with no previous ultrasound experience can be taught to perform the scan on patients with 3-h of intensive training [9]. However, for clinical evaluation a more rigorous approach to training is required.

The primary aim of this study was to evaluate the effectiveness of a structured 8-week training programme to teach the PAD-scan to healthcare professionals at the front line of diabetic foot care. A secondary aim was to evaluate the level of agreement between the PAD-scan, pulse palpation and audible handheld Doppler in assessing the arterial status of a diabetic foot.

## Participants and methods

The study was approved by the National Research Ethics Committee (reference no. 17/LO/1447) and forms the first stage of a future diagnostic evaluation study.

### Diabetic foot care teams

Diabetic foot care team members at a central London teaching hospital, and a district general hospital in London were invited to participate in the study as part of our research team. Five Podiatrists and one Consultant Diabetologist agreed to participate.

### Training programme overview

The training programme was divided into four phases over 8 weeks. The design of the programme was informed by the feedback received from our previous departmental experience of running ultrasound courses for novices [9]. All scans performed in this study utilised the Mindray M7 (Shenzhen, China) Portable Ultrasound System with a linear 6–14 kHz transducer.

### Phase 1: Intensive one-day training course

The day consisted of a standardised 45-min group didactic teaching session provided by a trained clinician. This covered relevant background information about arterial disease detection, basic DUS theory and the PAD-scan protocol. This was followed by 90-min of PAD-scan simulation training and 90-min of PAD-scan training on healthy medical student volunteers. Simulation training was performed using a high fidelity pulsatile flow simulator and a single straight vessel phantom (Axiom Medical Ltd., London, United Kingdom).

Participants were required to perform qualitative spectral waveform analysis and velocity measurements of random lower limb arterial waveforms generated by the simulator. Random waveforms generated demonstrated variation in waveform characteristics (monophasic, biphasic triphasic), peak systolic velocity (PSV), spectral broadening and the presence or absence of infilling of the spectral window. Spectral broadening (widening of the spectral waveform – or increased frequency spread) and the presence of infilling of the spectral window (or loss of systolic window) may suggest the presence of arterial disease [10].

Specific training was given for scanning heavily calcified, low flow, and occluded vessels. All practical training was delivered by a team of three vascular scientists in a 1:1 trainer to student ratio at the Vascular Laboratory at Hammersmith Hospital, London.

### Phase 2: Supervised patient scanning

Training was conducted in the respective diabetic foot clinics by a supervising vascular scientist with more than 10 years of clinical and teaching experience in vascular US (author CG). Training was pre-arranged for two half-day clinics per week at each site over a period of 5-weeks. On these days, Consecutive patients presenting to the respective diabetic foot clinics were asked to take part in the study and provide informed consent. Patients unable to provide informed consent were excluded. Scans were performed in addition to patients’ routine clinical workup. Patient demographic data, clinical details (presence of ulceration, ulcer severity score) and the results of the routine clinical workup (assessment for neuropathy, pulse palpation and/or hand held Doppler assessment) were recorded for later analysis. Peripheral neuropathy was assessed using a combination of 10 g monofilament testing at 10 separate sites on the foot, testing of vibration perception using a 128-Hz tuning fork and proprioception at the first metatarsophalangeal joint. Loss of sensation of any of these modalities was indicative of neuropathy.

Participants performed 15 supervised PAD-scans over 5-weeks. The supervising vascular scientist provided real time feedback to each participant. All scans were timed by a facilitator (total transducer-on-skin time) and assessed by the vascular scientist for technical performance using the previously validated Duplex Ultrasound Objective Structured Assessment of Technical Skills tool (DUOSATS) [11]. An abbreviated version of the DUOSATS assessment tool, with 7 domains scored on a Likert scale, was used because the last four domains focused on stenosis assessment and reporting, which were not relevant to the present study. This gave the DUOSATS tool a minimum and maximum attainable score of 6 and 26, respectively (Additional file 1: Figure S1).

After each scan the participants recorded their findings on a data collection sheet and rated their confidence level on a 5-point Likert scale *(1, really not confident; 2, not confident; 3, not sure; 4, confident; 5, really confident).* Participants gave a confidence rating for each of the two vessels scanned (i.e. anterior and posterior tibial arteries), giving a minimum and maximum confidence score of 2 and 10, respectively, for each scan.

Subsequently, the vascular scientist repeated the PAD-scan on the patient and recorded their findings on a data collection sheet. These assessments were not blinded because the vascular scientist was present and providing training during the participants attempt. Qualitative waveform interpretation *(*i.e. *waveform Character (monophasic, biphasic, triphasic, venous like flow or occlusion) and presence or absence of spectral broadening and infilling of the spectral window in biphasic waveforms only)* was later compared between the participant and the vascular scientist to calculate agreement. To allow us to gauge how technically challenging each scan was, the vascular scientist also classified the calcification of the vessel as either mild, moderate or severe *(mild = visible calcification, no acoustic shadowing; moderate = short segments of acoustic shadowing; severe = long segments of acoustic shadowing or occlusion).*

Generic technical feedback and selected anonymised waveforms with interpretative descriptions were provided weekly to participants during the supervised phase of training.

### Phase 3: Independent patient scanning

Participants were asked to independently perform 15 scans over 3-weeks. Consecutive patients presenting to the respective diabetic foot clinics were consented and asked to take part in the study. Patients unable to provide informed consent were excluded. Scans were performed in addition to patients’ routine clinical workup. Participants kept a logbook of scans and saved scan images on to the ultrasound machine. These were used to provide opportunistic individual feedback to participants. No assessments were completed during the independent scanning phase.

### Phase 4: Evaluation and review of performance

The breadth of participants’ experience as well as the level of their theoretical knowledge and technical skill were formally evaluated at the end of the 8-week training programme.

Theoretical knowledge regarding qualitative waveform interpretation was assessed by a 20-min exam, consisting of 20 multiple-choice questions (MCQs) with a pass mark of 70%.

Practical skills were assessed on a patient using the DUOSATS tool. A successful attempt was defined as being completed in less than 10-min with a minimum DUOSATS score of 23 out of 26 and perfect agreement with the vascular scientist qualitative PAD-scan assessment.

Additionally, logbooks of independent scans and all screenshots were reviewed to assess the breadth of experience and quality of independent scans. Participants were required to perform 15 supervised scans and 15 independent scans.

### Agreement between diagnostic modalities

For the purpose of assessing agreement between the PAD-scan, pulse palpation and hand held Doppler assessment, the following criteria for the presence of arterial disease were set:

- *PAD-scan:* presence of a monophasic signal, venous like slow flow or an occlusion in one or both vessels (i.e. anterior and posterior tibial arteries at the ankle) as per the vascular scientist assessment. For the purpose of descriptive analysis, possible characteristics of biphasic signals, which may suggest the presence of arterial disease, were also collected (presence of spectral broadening or infilling of the spectral window).
- *Pulse palpation:* absence of one or both foot pulses.
- *Hand held Doppler:* Presence of a monophasic signal or an absent signal in one or both vessels.

### Patient and participant feedback

Anonymised patient feedback was collected from those who agreed to be scanned during the supervised training phase. Patients rated their level of agreement *(completely disagree, mostly disagree, neither disagree nor agree, mostly agree or completely agree)* with various statements regarding the PAD-scan. Participants were also asked to provide feedback on the training programme after completion of the course. Additionally, participants’ attitudes towards the PAD-scan were explored by asking them to rate their level of agreement *(completely disagree, mostly disagree, neither disagree nor agree, mostly agree or completely agree)* with various statements.

### Statistical analysis

All statistical analyses were done using the Statistical Package for Social Sciences (SPSS) version 23 (IBM, Armonk, New York) software package. The Shapiro-Wilk test was used to assess for normalisation of data. DUOSATS score and time taken demonstrated non-Gaussian distribution and log transformation did not normalise the data. Therefore, Wilcoxon Signed-Rank Tests were used to assess the difference in values between scans and significance was set at *p* < 0.05. To assess the learning curve patterns, linear regression and curvilinear regression functions were fit onto the DUOSATS scores and time, to test whether the pattern of learning was linear or followed the shape of a curve.

Cohen’s Kappa statistic (κ) [12] was used to test for agreement between vascular scientist and participant waveform interpretation as well as agreement between the results of PAD-scan as compared to pulse palpation and hand held doppler assessment; κ was interpreted using the following: κ = 1.0, perfect agreement; κ > 0.80, very good agreement; 0.60 < κ ≤ 0.80, good agreement; 0.40<κ≤0.60, moderate agreement; 0.20<κ≤0.40, fair agreement; κ≤0.20, poor agreement. A four-point moving average was used to plot the graph for agreement and self reported confidence scores.

## Results

### Diabetic foot team demographics

Of the six diabetic foot team members who participated, two were male and four were female. Five of the participants had worked in their respective field for more than 5 years and one participant had worked in their field for less than 5 years. None of the participants had any previous ultrasound experience.

### Patient scanning and demographics

Participants completed a total of 90 PAD-scans in 65 patients during the supervised scanning phase. Each participant scanned 15 patients. No patients were scanned twice by the same participant.

Patient demographics are presented in Table 1. The majority of patients recruited had neuropathy and active foot disease. Additionally, 60% of the arteries scanned had either moderate or severe levels of calcification, representing technically challenging cases.

**Table 1:** The characteristics of the 65 patients who were scanned during the supervised scanning phase

	Patient Population
Gender, n (%)	
Male	46 (70.8%)
Female	19 (29.2%)
Age, mean (± S.D.)	67.7 (± 11.5)
Diabetes Type, n (%)	
T1DM	6 (9.2%)
T2DM	59 (90.8%)
Years with Diabetes, mean (± S.D.)	19.9 (± 11.2)
Co-morbidities, n (%)	
None	4 (6.2%)
Renal Failure	23 (35.4%)
Retinopathy	31 (47.7%)
Previous MI	10 (15.4%)
Angina	14 (21.5%)
Heart Failure	10 (15.4%)
Previous Stroke	10 (15.4%)
Hypertension	46 (70.8%)
Neuropathy, n (%)	45 (69.2%)
Active Ulcer, n (%)	52 (80%)
Duration of Active Ulcer, median (IQR) [days]	150 (40–360)
Active Ulcer SINBAD Score, median (IQR)	2 (2–3)
Severity of Arterial Calcification, n (%)	
Mild	62 (40.0%)
Moderate	50 (32.3%)
Severe	43 (27.7%)

*S.D* standard deviation, *IQR* interquartile range, *MI* myocardial infarction, *T1DM* type 1 diabetes mellitus, *T2DM* type 2 diabetes mellitus, *SINBAD* (site, ischemia, neuropathy, bacterial infection, area, depth) classification system for diabetic foot ulcers [20], components of the classification system can produce scores between 0 to 6 with increasing severity

### Performance during supervised training phase

#### Time

**Fig. 1:**
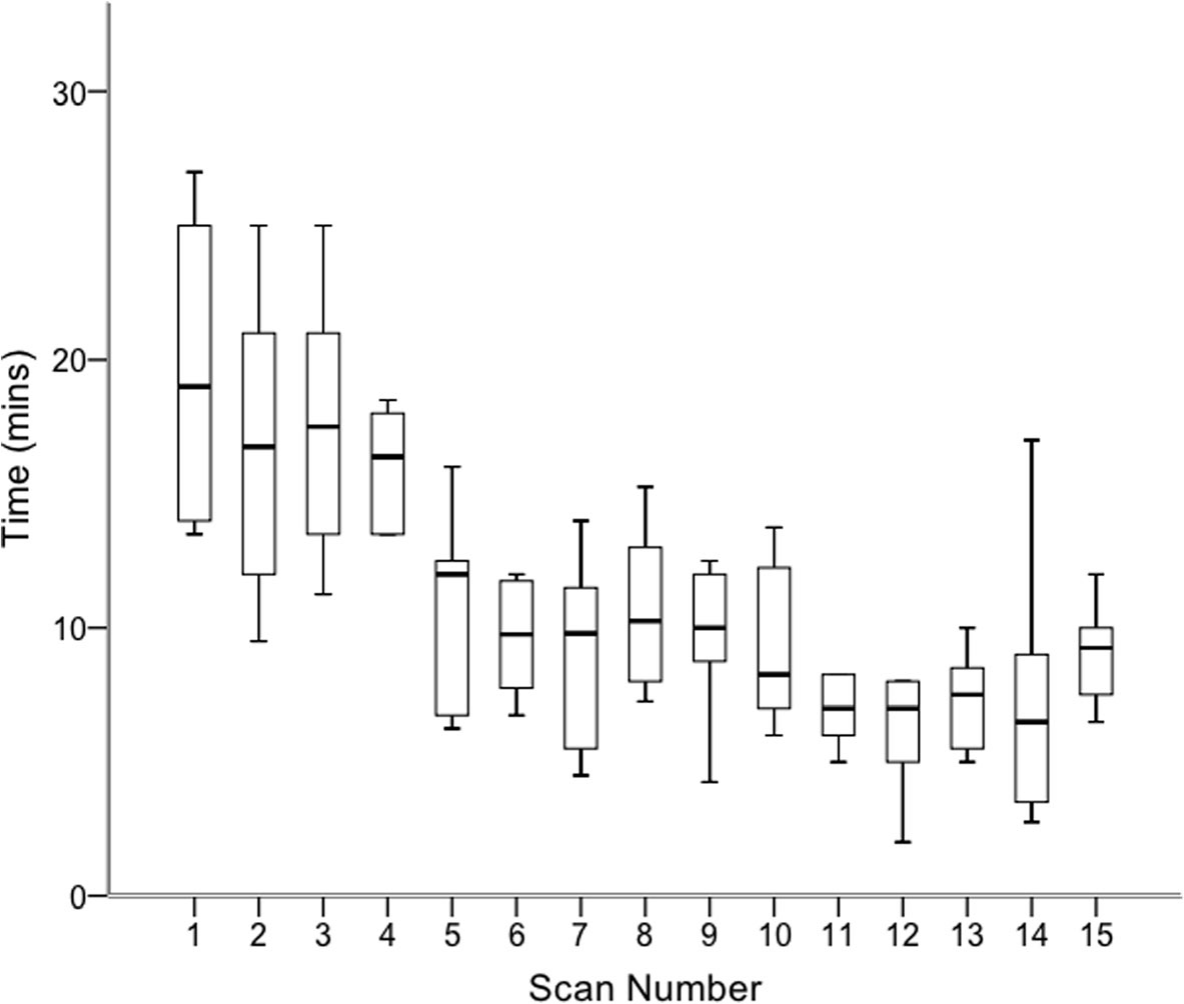
Boxplots of the time results from the supervised scanning phase

There was a statistically significant reduction in median time taken to perform each scan when comparing the initial and final attempts during the supervised training phase (19 min (IQR 13.9–25.5) vs 9.3 min (IQR 7.3–10.5)**;** *p* = 0.028); Fig. 1.

#### Duplex ultrasound objective structured assessment of technical skills (DUOSATS)

**Fig. 2:**
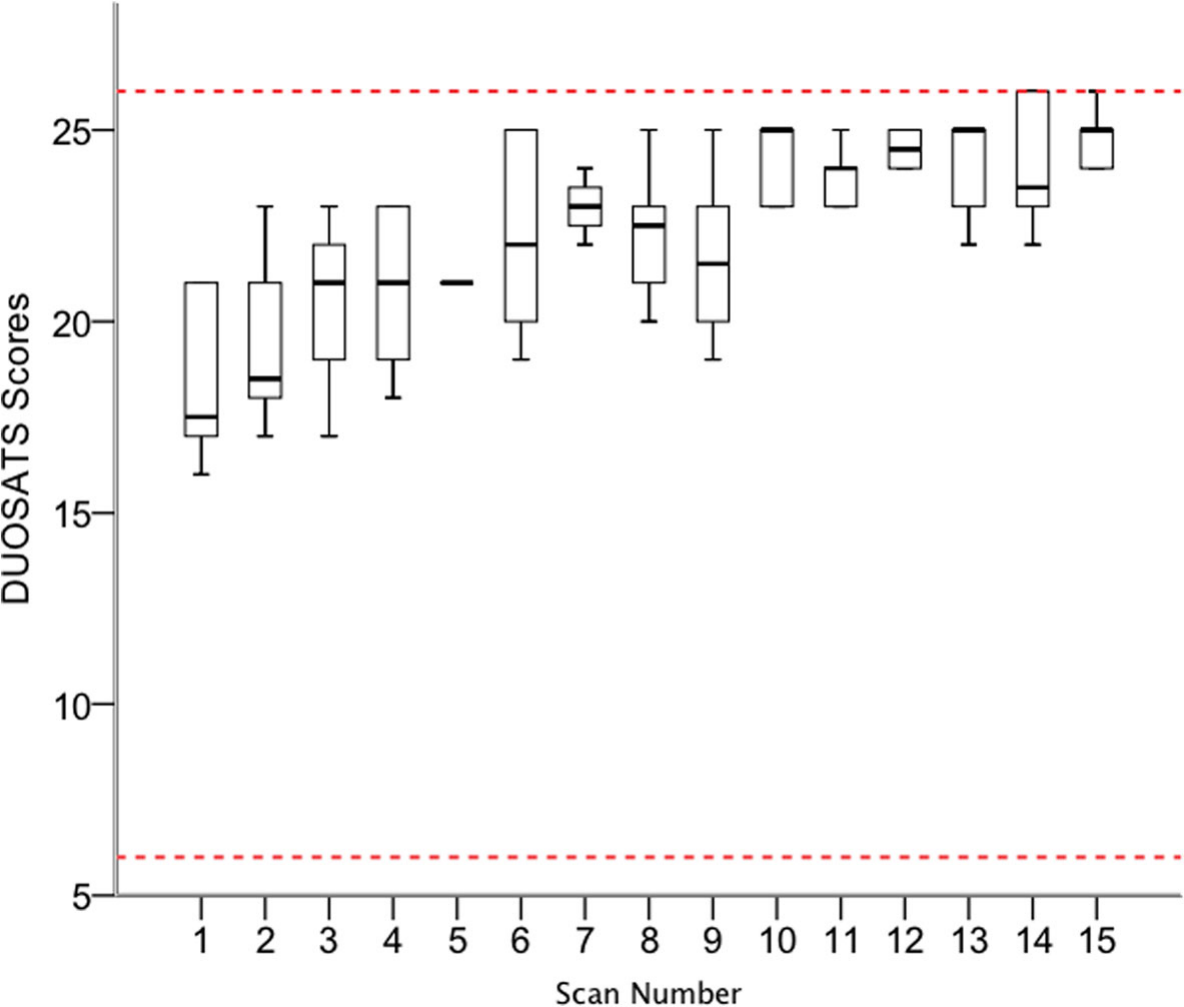
Boxplots of the Duplex ultrasound objective structured assessment of technical skills (DUOSATS) scores from the supervised scanning phase. The broken horizontal lines represent the minimum (6) and maximum (26) attainable scores

Similarly, there was a statistically significant improvement in median DUOSATS scores when comparing the initial and final attempts during the supervised training phase (17.5 (IQR 16.8–21) vs 25 (IQR 24–25.3); *p* = 0.027); Fig. 2.

#### Learning curve analysis

Curvilinear regression for both time and DUOSATS scores, demonstrated a higher coefficient of determination value compared to linear regression (Time R^2^; 0.48 vs 0.39 and DUOSATS R^2^ 0.55 vs 0.53 respectively) suggesting that curvilinear regression is a better fit to the data. Therefore, the pattern of learning followed more of a curve, such that learning occurred most rapidly in the beginning of the training programme and then slowed down thereafter.

#### Agreement

**Fig. 3:**
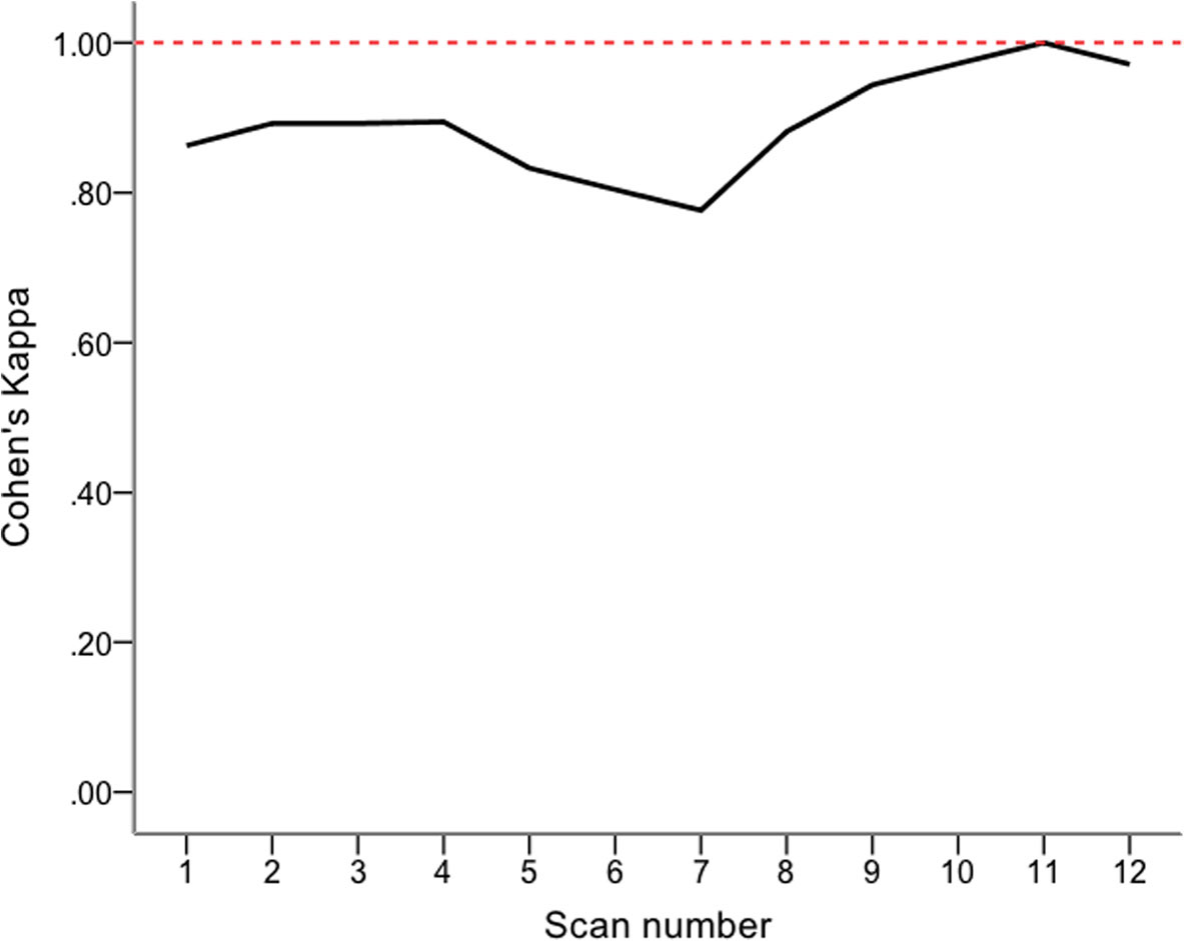
A 4-point moving average for Cohen’s Kappa (κ) is plotted against PAD-scan number. Perfect agreement (κ=1, represented by the broken horizontal line) is reached by scan 11

The four-point moving average of κ demonstrates that ‘perfect agreement’ (κ=1) in waveform interpretation was reached between the supervising vascular scientist and the participants by scan 11. Before scan 11 there was either ‘good’ or ‘very good’ agreement (Fig. 3).

#### Confidence

**Fig. 4:**
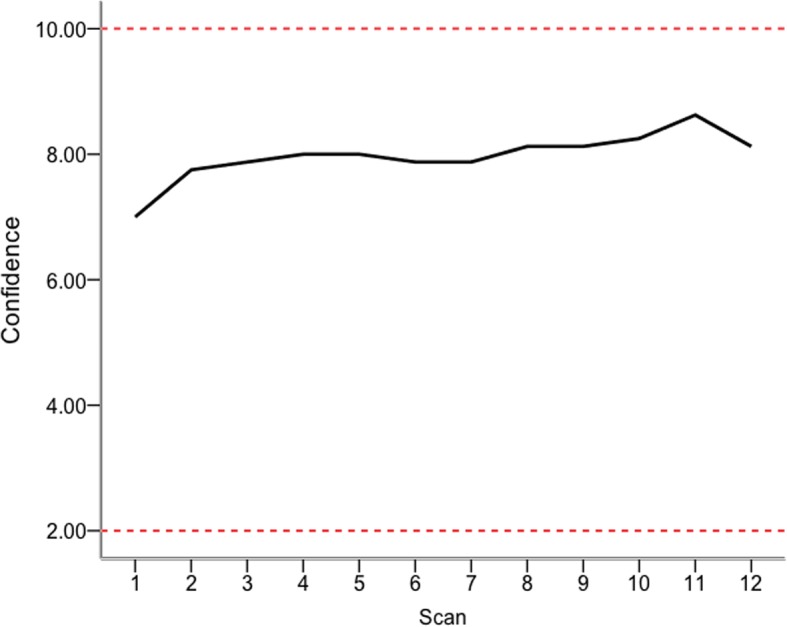
A 4-point moving average for participant confidence plotted against PAD-scan number. Participants gave a confidence rating for each vessel, giving a minimum and maximum confidence score of 2 and 10, respectively, for each scan

When considering self rated confidence (really not confident, 2; really confident, 10), four-point moving average scores increased steadily through the supervised scanning phase, reaching a peak of 8.6 at scan 11 (Fig. 4).

### Final evaluation of performance

All participants passed the MCQ knowledge exam (mark range, 75–85%), completed scans in the required time (median 5.4 min, IQR 5.3–5.9), attained the required DUOSATS scores (median 26, IQR 26–26) and achieved perfect agreement with the vascular scientist (κ=1). Self-rated confidence was also high amongst participants (median 8.5, IQR 8–9.8).

### Agreement between the diagnostic modalities

Using predefined criteria for the presence of arterial disease (***PAD-scan,*** presence of a monophasic signal, venous like slow flow or an occlusion in one or both vessels; ***pulse palpation,*** absence of one or both foot pulses; ***hand held Doppler,*** presence of a monophasic signal or an absent signal in one or both vessels) arterial disease was detected in 68.1% of all assessments (*n* = 62/90) using the PAD-scan, 44.3% of cases using pulse palpation (*n* = 31/70) and 75.6% using the handheld Doppler (n = 31/41). The PAD-scan had ‘fair agreement’ with pulse palpation (κ=0.315, *p* = 0.003), and ‘moderate agreement’ with handheld Doppler (κ=0.552, *p* < 0.001) for the assessment of arterial disease.

For each separate vessel scanned further analysis of agreement between the PAD-scan and hand held Doppler waveform assessment was conducted (i.e. whether the waveform was monophasic, biphasic, triphasic or absent). This demonstrated ‘fair’ to ‘poor’ agreement (κ=0.252, p < 0.001) between the PAD-scan *(n = 180; monophasic 45%, biphasic 23%; triphasic 18%, absent signal 14%)* and handheld Doppler assessments *(n = 82; monophasic 66%, biphasic 23%; triphasic 7.3%, absent signal 3.7%).*

We performed further analysis evaluating the effect of applying criteria for abnormal features of biphasic waveforms (i.e. the presence of spectral broadening and/or infilling of the spectral window), as detected using the PAD-scan, on arterial disease detection. This resulted in arterial disease being detected in 85.7% (*n* = 79/90) of all patient assessments using the PAD-scan.

### Patient and participant feedback

#### Patient feedback

**Fig. 5:**
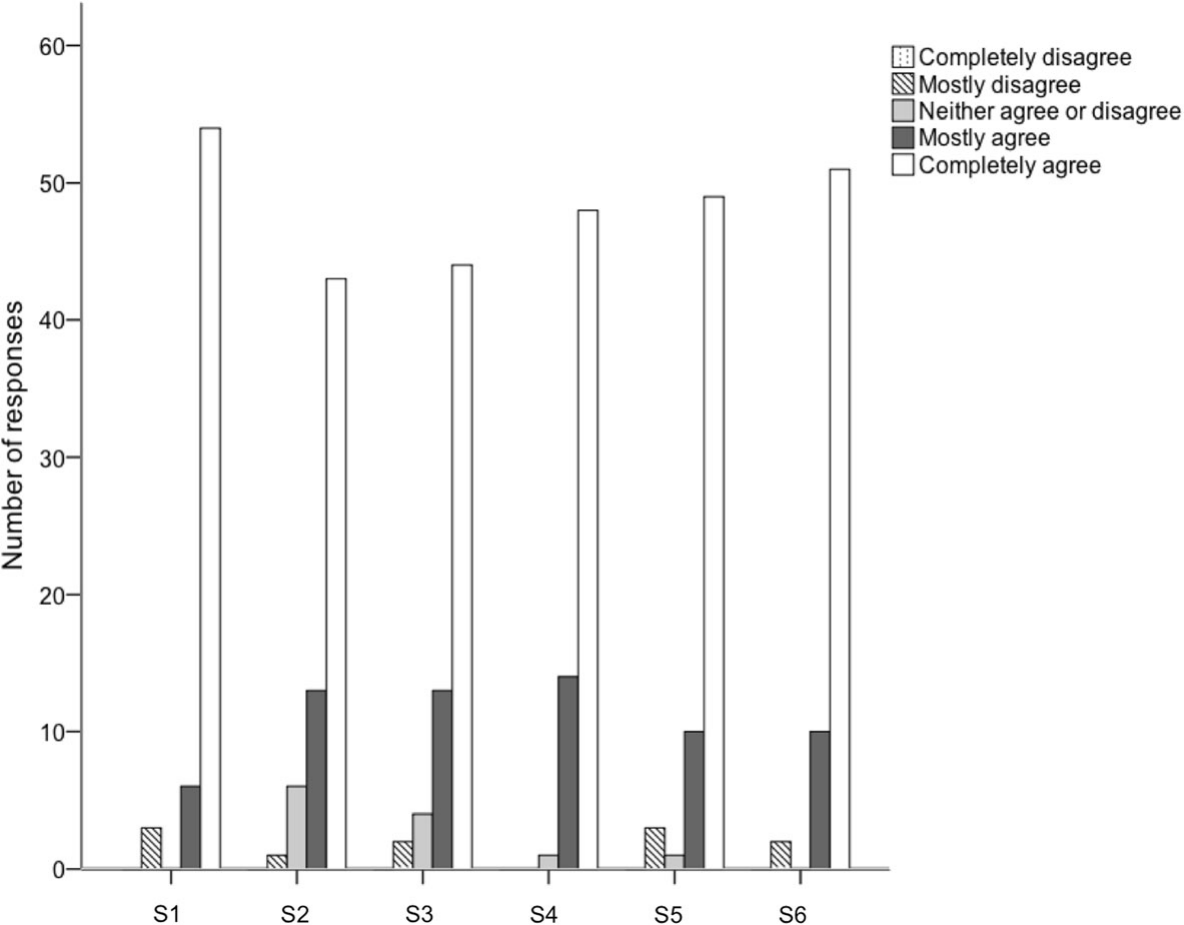
Bar chart representing patient responses *(completely disagree, mostly disagree, neither disagree nor agree, mostly agree or completely agree)* with the following six statements: ***S1.*** ‘I felt comfortable during the scan’. ***S2.*** ‘I feel that this scan should be performed routinely in diabetic foot assessment’. ***S3.*** ‘I feel that podiatrists trained in performing the PAD-scan are better qualified to detect poor circulation’. ***S4.*** ‘I would want my feet looked after by a podiatrist who can perform the PAD-scan’. ***S5.*** ‘I feel more confident in the assessment I had today’ ***S6.*** ‘I feel more satisfied with the assessment I had today’

Sixty-three out of 65 patients provided feedback regarding the PAD-scan **(**Fig. 5**)**; 95% of patients reported feeling comfortable during the scan, 94% of patients felt confident and 97% of patients felt satisfied with their assessment.

#### Participant feedback

Participants were asked for feedback regarding both the training programme and the PAD-scan.

Regarding the training programme all six participants either agreed or mostly agreed that the length of the training programme was appropriate, the intensive 1 day training course was beneficial, supervised scanning in clinic was beneficial and that the ultrasound equipment was easy to use and easily portable.

Feedback was also positive when participants were asked to rate (*1, completely disagree; 2, mostly disagree; 3, neither disagree nor agree; 4, mostly agree; 5, completely agree)* their level of agreement with various statements regarding the potential application and implications of the PAD-scan in clinical practice: I think the PAD-scan will help with ulcer healing times (mean 4, SD ± 1.5); will improve the efficiency of local debridement’s (3.8 ± 1.6); will help with triaging patients for further assessment (4.8 ± 0.4); is a useful tool for detecting arterial disease in diabetic patients (4.8 ± 0.4); can eliminate the need to perform other non-invasive screening tests for arterial disease (3.8 ± 1.8).

## Discussion

In this study we have evaluated the effectiveness of an 8-week training programme to teach the PAD-scan to front line healthcare workers. As demonstrated in our previous work [9], participants attained basic PAD-scan skills after an intensive training course on day 1 with 3-h of practical training. However, as expected further substantial improvements were seen in time taken to perform scans, DUOSATS scores (surrogate marker of technical skill) and agreement with vascular scientist assessments (surrogate marker of accuracy) during the supervised scanning phase. As demonstrated by the pattern of improvement, learning was rapid initially and slowed towards the end of the supervised scanning phase. This was followed by a period of supported independent scanning, allowing for participants to transition into confidently scanning patients on their own. The effectiveness of this approach is demonstrated in the excellent results of the final evaluation of performance during which all scans were performed accurately, to a high technical standard and in 5-min.

The effectiveness of the training programme is further reflected in the positive feedback received by the participants as well as their high self-reported confidence scores. The programme proved feasible to run and integrated well into both diabetic foot clinics with minimal disruption to clinical workflow. This could be readily replicated in its current format in other centers.

Patient feedback was also positive with high reported levels of confidence and satisfaction in their clinical assessment. This is particularly important as patients with diabetic complications have been reported to have overall lower treatment satisfaction and higher levels of anxiety [13, 14].

In this training programme, learning was achieved despite training taking place in two hospital clinics looking after people with complex, active diabetic foot disease. The majority of patients recruited had a long duration of foot ulceration in the presence of peripheral neuropathy. Neuropathy has been demonstrated to make the detection of arterial disease considerably more difficult in the diabetic foot, with poor sensitivity of bedside tests in this group of patients [15]. Furthermore, a large proportion of patients in our cohort were found to have moderate to high levels of arterial calcification, supporting the notion that they represent a technically challenging group of patients. This may explain the disappointing levels of agreement seen between the PAD-scan, pulse palpation and hand held Doppler assessments. In the absence of a reference standard, such as a full lower limb DUS, it is not possible to determine which of these tests demonstrated superior accuracy. However, the low levels of agreement between them does suggest that there is a difference present.

The PAD-scan has a number of potential advantages over current bedside tests used to detect the presence of arterial disease in people with diabetes. It allows for direct visualisation of the tibial vessels and allows for selective sampling of Doppler signals from a region of interest. This may reduces the chance of mistakenly sampling a nearby collateral, which could be misleading, but also allows the operator to avoid calcification in the vessel wall and gain more granular Doppler waveform analysis. Another potential advantage of the PAD-scan is that it can provide more detailed qualitative waveform information. This may be particularly useful for more detailed assessment of biphasic signals. Although these are commonly considered a normal consequence of aging, there is likely to be a spectrum of normal and abnormal biphasic signals [16, 17]. Features such as spectral broadening, infilling of the spectral window and long forward flow in diastole have been described but warrant further investigation to evaluate their clinical relevance [10]. The PAD-scan also has a number of potential disadvantages that need to be considered including equipment costs and the availability of skilled vascular scientists to support training.

The presence of arterial disease is an important risk factor in the presence of a diabetic foot ulcer and evidence suggests that revascularisation must be performed with minimal delay in order to achieve the best possible outcomes [2]. This represents an important target for improving ulcer care but has not been well addressed in the literature. Root cause analysis of 140 amputations in Sheffield (United Kingdom) identified that in 30 (21%) cases amputations were the result of delayed vascular referral, investigation or intervention [18]. If an accurate bedside test to detect arterial disease were available then this would allow for rapid triage of those requiring urgent revascularisation. The PAD-scan has a theoretical advantage over other bedside tests in detecting arterial disease and we have demonstrated that it can be readily learned as part of a training programme integrated into current clinic setups.

However, caution should be exercised before integrating the PAD-scan into clinical practice. The diagnostic criteria for qualitative waveform analysis using the PAD-scan has not been established and one must be vigilant that normal appearing waveform in a focused scan may not be entirely sufficient as a rule out test for peripheral arterial disease [19]. Therefore, the diagnostic accuracy of the PAD-scan as compared to other bedside tests in detecting arterial disease in diabetes warrants further evaluation as part of a diagnostic accuracy study. Furthermore, ultrasound is a user-dependent technology and although establishing an effective training programme is the first step to ensure a high quality of care, users will also have to provide evidence of ongoing practical experience for accreditation and would also be required to attend additional revision sessions to demonstrate continued professional development. Quality assurance measures such as regular audits of scan accuracy may also be required.

### Limitations

This work has a number of limitations. Assessments were not blinded and were performed by a single assessor who was also responsible for training participants. Furthermore, routine clinical examination was not standardised, as this was left to the discretion of the clinical teams, resulting in missing data for pulse palpation and handheld Doppler assessments. However, evaluation of agreement between these and the PAD-scan was only a secondary aim of this study.

## Conclusion

A structured training programme integrated into diabetic foot clinics is effective in teaching the PAD-scan to ultrasound-novice healthcare professional at the front line of diabetic foot care. Despite the technically challenging caseload, participants demonstrated that the PAD-scan can be performed rapidly and to a high technical standard and accuracy. Further work is required to evaluate the diagnostic accuracy of the PAD-scan to other bedside tests.

## Additional file

Additional file 1 : **Figure S1**. The full DUOSATS assessment tool. The last four domains (grey) concern stenosis assessment and reporting, which are not relevant to the present study. Therefore, these domains were excluded from the assessment, giving a minimum and maximum attainable DUOSATS score of 6 and 26, respectively. (PNG 558 kb)

## Abbreviations

ABPI: Ankle brachial pressure indices
DUOSATS: *Duplex Ultrasound Objective Structured Assessment of Technical Skills tool*
DUS: Duplex ultrasound
PAD-scan: Podiatry Ankle Duplex Scan
PSV: Peak systolic velocity
TBPI: Toe brachial pressure indices
TCO_2_: Transcutaneous tissue oxygen
US: Ultrasound
