# Supplementary figures and images for: Effectiveness of a multicenter training programme to teach point-of-care vascular ultrasound for the detection of peripheral arterial disease in people with diabetes
Source: J Foot Ankle Res. 2018 Jul 16;11:41. doi: 10.1186/s13047-018-0283-0 (PMC6048877; doi:10.1186/s13047-018-0283-0)

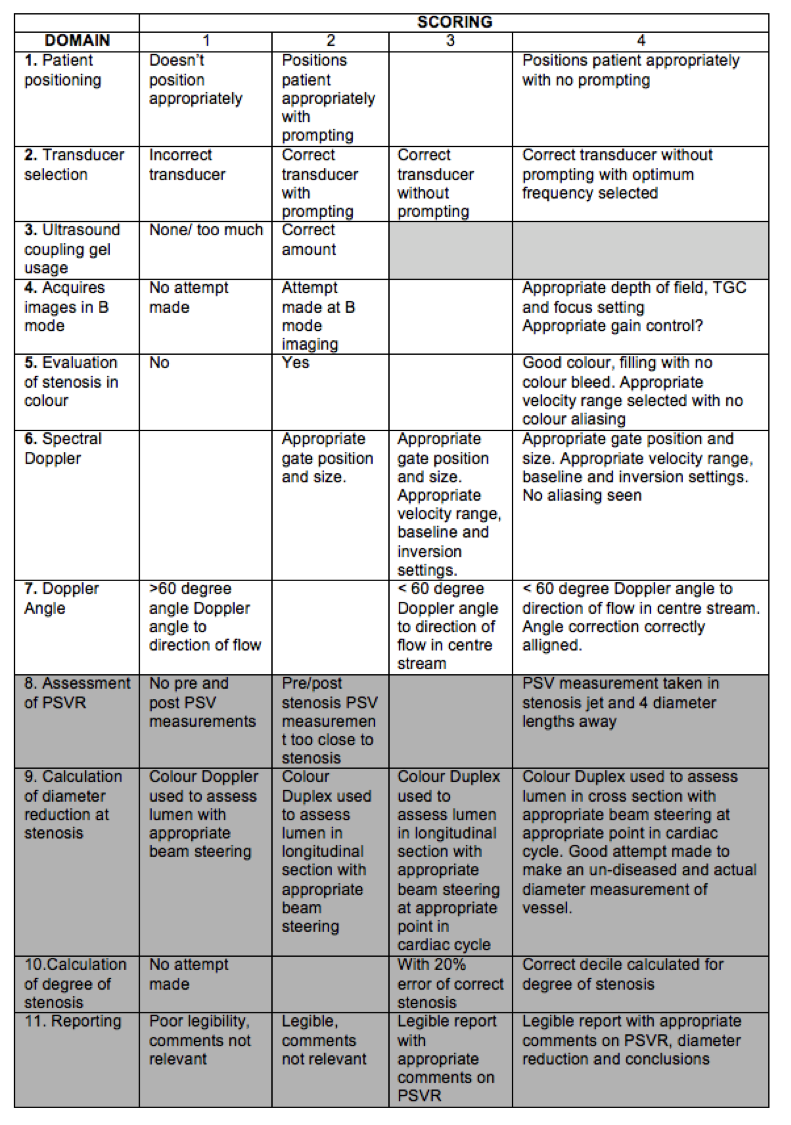

Supplement: Supplementary file 1 — : Figure S1. The full DUOSATS assessment tool. The last four domains (grey) concern stenosis assessment and reporting, which are not relevant to the present study. Therefore, these domains were excluded from the assessment, giving a minimum and maximum attainable DUOSATS score of 6 and 26, respectively. (PNG 558 kb) [file 13047_2018_283_MOESM1_ESM.png]
